# Trelagliptin (SYR-472, Zafatek), Novel Once-Weekly Treatment for Type 2 Diabetes, Inhibits Dipeptidyl Peptidase-4 (DPP-4) via a Non-Covalent Mechanism

**DOI:** 10.1371/journal.pone.0157509

**Published:** 2016-06-21

**Authors:** Charles E. Grimshaw, Andy Jennings, Ruhi Kamran, Hikaru Ueno, Nobuhiro Nishigaki, Takuo Kosaka, Akiyoshi Tani, Hiroki Sano, Yoshinobu Kinugawa, Emiko Koumura, Lihong Shi, Koji Takeuchi

**Affiliations:** 1 Enzymology and Biophysical Chemistry, Takeda California, Inc., San Diego, California, United States of America; 2 Computational Sciences and Crystallography, Takeda California, Inc., San Diego, California, United States of America; 3 Cardiovascular and Metabolic Drug Discovery Unit, Pharmaceutical Research Division, Takeda Pharmaceutical Company Limited, Fujisawa, Kanagawa, Japan; 4 Bio-Molecular Research Laboratories, Pharmaceutical Research Division, Takeda Pharmaceutical Company Limited, Fujisawa, Kanagawa, Japan; 5 Takeda Development Center Japan, Takeda Pharmaceutical Company Limited, Osaka, Japan; National Research Council of Italy (CNR), ITALY

## Abstract

Trelagliptin (SYR-472), a novel dipeptidyl peptidase-4 inhibitor, shows sustained efficacy by once-weekly dosing in type 2 diabetes patients. In this study, we characterized *in vitro* properties of trelagliptin, which exhibited approximately 4- and 12-fold more potent inhibition against human dipeptidyl peptidase-4 than alogliptin and sitagliptin, respectively, and >10,000-fold selectivity over related proteases including dipeptidyl peptidase-8 and dipeptidyl peptidase-9. Kinetic analysis revealed reversible, competitive and slow-binding inhibition of dipeptidyl peptidase-4 by trelagliptin (t_1/2_ for dissociation ≈ 30 minutes). X-ray diffraction data indicated a non-covalent interaction between dipeptidyl peptidase and trelagliptin. Taken together, potent dipeptidyl peptidase inhibition may partially contribute to sustained efficacy of trelagliptin.

## Introduction

An estimated 347 million people worldwide have diabetes mellitus (diabetes) [1]. Type 2 diabetes (T2DM), a metabolic disease characterized by hyperglycemia caused by impaired insulin secretion (beta cell dysfunction) and insulin resistance of peripheral tissues, represents around 90% of diagnosed diabetes cases. Causal factors include diet, obesity, genetics, and physical inactivity. An estimated 1.5 million deaths were directly caused by diabetes in 2012, and more than 80% of diabetes deaths occur in low- and middle-income countries. Projections for the prevalence of the disease vary and the World Health Organization predicts a 50% increase in cases worldwide in the next decade.

Complications from diabetes include nephropathy, neuropathy, retinopathy, and macrovascular diseases such as stroke or heart disease, and diabetes is a leading cause of blindness, amputation and kidney failure. T2DM is initially managed by life style modifications like increasing exercise and making dietary changes. However, medication may become necessary if these approaches do not lower blood glucose levels sufficiently. As noted elsewhere [2], improving a patient’s adherence to medication during long-term treatment is important in order to maintain favorable glycemic control, which may prevent the onset or lessen the severity of diabetic complications.

Dipeptidyl peptidase-4 (DPP-4), the serine protease responsible for metabolism of the incretin hormones glucagon-like peptide-1 and glucose-dependent insulinotropic polypeptide, plays an important role in regulating glucose homeostasis [3]. Thus, DPP-4 is an attractive target for therapeutic intervention, and inhibitors of DPP-4 have been shown to be an effective therapy for treatment of T2DM [4].

Trelagliptin (SYR-472, zafatek) is a novel once-weekly DPP-4 inhibitor approved in Japan. While other current marketed DPP-4 inhibitors are dosed once or more than once per day, trelagliptin showed efficacy as well as a suitable safety profile in T2DM patients by dosing once-weekly in a clinical trial setting [2, 5]. In this article, we characterized the *in vitro* profile of this unique once-weekly DPP-4 inhibitor, trelagliptin, and examined its contribution to sustained efficacy in the treatment of T2DM.

## Materials and Methods

### Chemicals

Alogliptin (2-[6-(3(R)-aminopiperidin-1-yl)-3-methyl-2,4-dioxo-3,4-dihydro-2H-pyrimidin-1-ylmethyl]benzonitrile), trelagliptin (2-[6-(3(R)-aminopiperidin-1-yl)-3-methyl-2,4-dioxo-3,4-dihydro-2H-pyrimidin-1-ylmethyl]-4-fluorobenzonitrile), and sitagliptin ((2R)-4-oxo-4-[3-(trifluoromethyl)-5,6-dihydro[1,2,4]triazolo[4,3-a]pyrazin-7(8H)-yl]-1-(2,4,5-trifluorophenyl)butan-2-amine) were synthesized at Takeda Pharmaceutical Company Limited.

### Animals

Male Sprague–Dawley rats (7 weeks old, n = 30) and male beagle dogs (2–5 years old, n = 3) were purchased from CLEA Japan (Tokyo, Japan) and Kitayama Labes Co., Ltd. (Nagano, Japan), respectively. All animals were housed in cages in a room with controlled temperature (23°C), humidity (55%) and lighting (lights on from 07:30 am to 07:30 pm) and were maintained on a laboratory chow diet (CE2 [CLEA Japan] for rats and DS-5 [Oriental Yeast Co., Ltd.] for dogs). Rat blood samples were taken from abdominal vein under ether inhalation anesthesia in anesthetized rats before euthanasia by exsanguination. Dog blood samples were collected from anterior limb veins in conscious dogs that were restrained in a retainer. After the blood collection, dogs were returned to their normal housing for other experiments. Plasma samples were prepared and stored at -80°C before use. The care and use of the animals and the experimental protocols used in this research were approved by the Experimental Animal Care and Use Committee of Takeda Pharmaceutical Company Limited.

### Human plasma samples

Human plasma was prepared from fresh blood of healthy volunteers who gave written informed consent. The measurement of DPP-4 activity using this human plasma was approved by the research ethics committee in Takeda Pharmaceutical Company Limited.

### Enzyme inhibition assays

Human DPP-4 enzyme used in these studies was obtained from several sources. Human DPP-4 partially purified from Caco-2 cells purchased from the ATCC (ATCC No. HTB-37; www.atcc.org), as described previously [6], was used to confirm trelagliptin inhibitor potency. For comparison among the DPP-4 inhibitors, trelagliptin, alogliptin and sitagliptin, commercially available recombinant human DPP-4 (Abnova, Taiwan) was used. For detailed kinetic studies, recombinant human DPP-4 was cloned, expressed and purified as described previously [7]. In addition, inhibition of plasma DPP-4 activity was determined using plasma samples of humans, dogs, and rats. The DPP-4 related proteases, dipeptidyl peptidase-2 (DPP-2) and prolyl endopeptidase (PEP), were prepared from rat kidney and brain, respectively, according to the method previously reported [8, 9]. Human dipeptidyl peptidase-8 (DPP-8), dipeptidyl peptidase-9 (DPP-9), and fibroblast activation protein α (FAPα) were purified by affinity chromatography from 293-F cells expressing each FLAG-tagged protein.

DPP-4 activity from Caco-2 cells or plasma was assayed using the chromophoric substrate Gly-Pro-*p*-nitroaniline (GP-pNA) (0.5 mmol/L final concentration) and carried out in pH 7.5 buffer containing 100 mmol/L Tris-HCl, 1 mg/mL bovine serum albumin, and 0.5 mg/mL CHAPS (3-[(3-cholamidopropyl)dimethylammonio]-1-propanesulfonic acid) for 60 min at 37°C (DPP-4 fraction from Caco-2 cells) or 30°C (plasma). Change in absorbance at 405 nm was measured to determine the reaction rate. Recombinant human DPP-4 (Abnova, Taiwan) activity was assayed using the fluorescent substrate Gly-Pro-7-amido-4-methyl-coumarin (GP-AMC) (90 μmol/L final concentration) and carried out in pH 7.8 buffer containing 25 mmol/L HEPES, 140 mmol/L NaCl, 1 mg/mL bovine serum albumin for 15 min at 37°C. The reaction was stopped by the addition of 100 μL of 25% (v/v) acetic acid, and fluorescence was measured (380 nm excitation/460 nm emission) using Envision 2103 Multilabel Reader (Perkin Elmer Japan, Japan). Reaction conditions for measurement of DPP-2, DPP-8, DPP-9, PEP, and FAPα activities are described in Table 1. Change in absorbance at 405 nm was measured to determine the reaction rate.

**Table 1:** Reaction conditions for measurement of DPP-2, DPP-8, DPP-9, PEP, and FAPα activities.

Enzymes	DPP-2	DPP-8	DPP-9	PEP	FAPα
Assay buffer*	100 mmol/L DMGA buffer(pH 5.5)	100 mmol/L Tris-HCl buffer(pH 7.5)	100 mmol/L Tris-HCl buffer(pH 7.5)	25 mmol/L glycine,25 mmol/L acetic acid, 23 mmol/LMES,75 mmol/L Tris, and100 mmol/L NaCl(pH 8.0)	88 mmol/L Na-K phosphate buffer(pH 7.0)
Substrates*	0.5 mmol/L Lys-Ala-pNA	1 mmol/L Gly-Pro-pNA	2 mmol/L Gly-Pro-pNA	2 mmol/L Ala-Pro-pNA	0.5 mmol/L Suc-Ala-Pro-pNA
Trelagliptin *	0.03, 0.1, 0.3, 1, 3, 10, 30 and 100 μmol/L				
Temperature and duration	37°C, 60 min	37°C, 90 min	37°C, 90 min	37°C, 60 min	37°C, 60 min

*Final concentrations are listed.

For detailed kinetic studies, GP-pNA was used as substrate and assays carried out in pH 7.4 buffer containing 20 mmol/L HEPES, 20 mmol/L MgCl_2_, 0.1 mg/ml bovine serum albumin, and 1% (v/v) DMSO at room temperature. In most cases, DPP-4 enzyme (1 nmol/L final concentration) was added last to initiate the enzymatic reaction, except when measuring the recovery of DPP-4 enzyme activity from a preformed DPP-4-inhibitor complex, in which case enzyme was first pre-incubated with trelagliptin for 70 min before initiating the reaction by dilution 50-fold into a reaction buffer containing a large excess (2 mmol/L, ca. 17x Km) of GP-pNA substrate. All assays were conducted as duplicates in 96-well format with total assay volume of 200 uL and absorbance at 405 nm was measured every 10 seconds to determine the reaction time-course. In most cases, the entire reaction progress curve was analyzed as described below. However, for initial rate studies to establish GP-pNA substrate-competitive inhibition by trelagliptin, only absorbance measurements from the first 40 seconds were used.

### Slow Binding Inhibition Model

**Fig 1:**
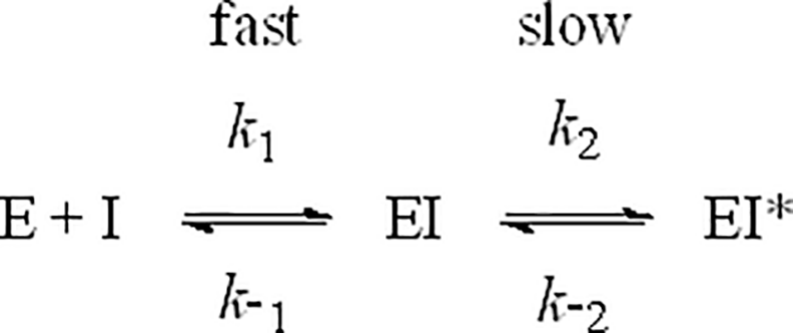
Two-step inhibition model.

Fig 1 illustrates a simple two-step inhibition model in which the inhibitor I binds to enzyme E in a rapid first step to form a weak EI complex with dissociation constant K_i_ (Eq 1), followed by a slower second step to yield an EI* complex, where inhibitor is bound more tightly, the overall dissociation is now given by K_i_* (Eq 2), and K_isom_ (Eq 3) is the equilibrium constant for conversion between weak EI and tight EI* complexes, respectively. The observed rate constants for association (k_on_) and dissociation (k_off_) of inhibitor from the tight EI* complex in Fig 1 are then given by Eqs 4 and 5, respectively.

$$K_{i}=\frac{E•I}{EI}=\frac{k_{-1}}{k_{1}}$$ (1)

$$K_{i}*=\frac{E•I}{\left(EI+EI*\right)}=\frac{K_{i}}{\left(1+K_{isom}\right)}\approx \frac{K_{i}}{K_{isom}}(when\;k_{2}>>k_{-2})$$ (2)

$$K_{isom}=\frac{k_{2}}{k_{-2}}$$ (3)

$$k_{\text{on}}\;(\text{onset of inhibition})\approx (k_{2}/K_{i})$$ (4)

$$k_{\text{off}}\;(\text{recovery of activity})\approx k_{-2}$$ (5)

### Kinetic analysis

To obtain estimates for the association (k_on_) and dissociation (k_off_) rate constants for trelagliptin inhibition of DPP-4, reaction progress curve data monitoring the onset of inhibition (DPP-4 enzyme added last to initiate reaction) were analyzed using the procedures outlined by Morrison [10]. Briefly, each progress curve was fitted to Eq 6:

$$A=v_{s}t+[{(v}_{o}-v_{s})(1-e^{-k^{\prime }t})/k\prime ]+A_{o}$$ (6)

where *t* is time, *A* is absorbance, and *A*_*0*_ is absorbance at time t = 0, to determine k', the apparent rate constant for decay from the faster initial rate (v_o_) to the slower inhibited steady-state rate (v_s_). An estimate for k_on_’, the apparent on-rate for trelagliptin binding, was obtained as the slope of the plot of k’ versus trelagliptin concentration according to Eq 7:

$$k^{\prime }=k_{\text{off}}+k_{\text{on}}\prime \;[\text{trelagliptin}]$$ (7)

The y-intercept of this plot yielded an estimate for k_off_, and thus an estimate of t_1/2_ (= (ln2/k_off_)) for dissociation of trelagliptin. To obtain an estimate for k_on_, Eq 8 was used to correct for the GP-pNA substrate concentration because trelagliptin is a substrate-competitive DPP-4 inhibitor:

$$k_{\text{on}}=k_{\text{on}}\prime \;(1+\left[\text{GP-pNA}\right]/{\text{K}}_{\text{m}})$$ (8)

The ratio (k_off_/k_on_) yielded a value for K_i_*, the steady-state inhibition constant for the tight EI* complex. K_i_* was also estimated by plotting 1/*v*_s_, where *v*_*s*_ is the steady-state rate at long reaction times, versus inhibitor concentration in a standard Dixon plot [11].

To obtain a more accurate estimate for k_off_, reaction progress curve data monitoring the recovery of DPP-4 enzyme activity following dilution of a preformed DPP-4- inhibitor complex into a reaction mixture containing a large excess of GP-pNA substrate were again fitted to Eq 6 to determine k’, the apparent rate constant for recovery from the slower inhibited initial rate (v_o_) to the faster steady-state rate (v_s_). As above, the y-intercept of the plot of k’ versus trelagliptin concentration according to Eq 7, yielded an estimate for k_off_.

### X-ray diffraction data

Wild-type human DPP-4 was purified and crystallized as previously reported [7, 12]. All protein-inhibitor complexes were obtained by soaking preformed DPP-4 crystals in a solution containing the compound of interest. Crystals were then cryo-protected with ethylene glycol and flash frozen in liquid nitrogen. X-ray diffraction data were collected at the Advanced Light Source (ALS) beam line 5.0.3, and processed using the program HKL2000 [13]. The structures of DPP-4 inhibitor complexes were determined by molecular replacement using MOLREP, utilizing the previously determined coordinates of DPP-4 with accession code 1R9M [7, 14]. Subsequent structure refinement and model building were performed utilizing REFMAC and XtalView [14, 15]. Bound inhibitors were clearly visible in the electron density maps.

## Results

### Potency of DPP-4 inhibition and specificity towards DPP-4-related proteases

**Fig 2:**
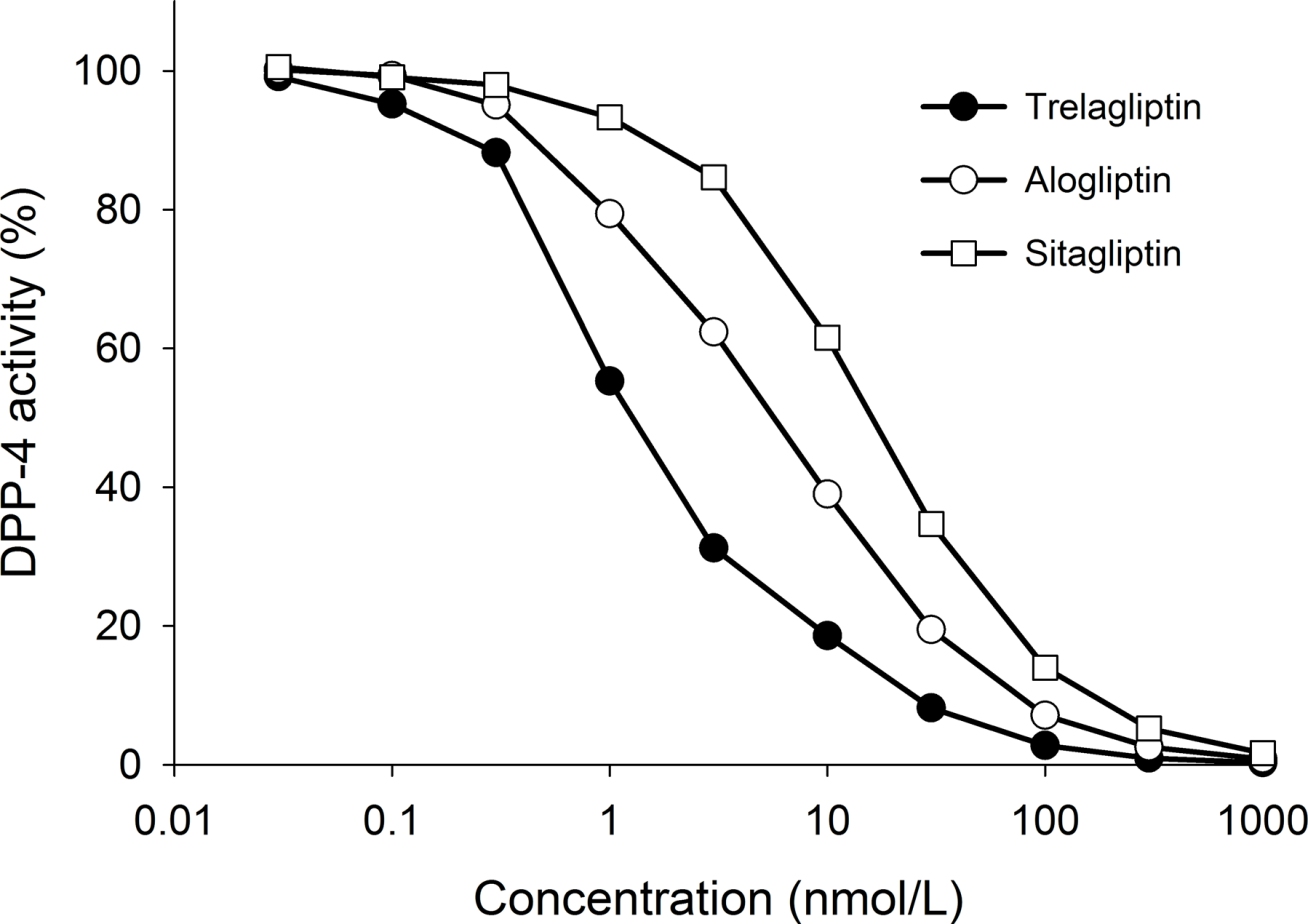
Concentration response curves of DPP-4 inhibitory activities by trelagliptin, alogliptin and sitagliptin. Concentration response curves of DPP-4 inhibitory activities by trelagliptin, alogliptin and sitagliptin. Activity was measured as described under Materials and Methods.

Trelagliptin exhibited potent inhibitory activity toward DPP-4 prepared from Caco-2 cells with an IC_50_ value of 5.4 nmol/L [95% confidence interval (CI) = 5.2–5.7]. Trelagliptin also inhibited human, dog, and rat plasma DPP-4 activity with IC_50_ values of 4.2 (CI = 4.1–4.3), 6.2 (CI = 6.0 –6.4), and 9.7 (CI = 8.0–11.8) nmol/L respectively (Table 2). When tested under identical experimental conditions using recombinant human DPP-4, trelagliptin exhibited potent inhibition with IC_50_ values for trelagliptin, alogliptin, and sitagliptin of 1.3 (CI = 1.1–1.5), 5.3 (CI = 5.0 –5.7), and 16.0 (CI = 15.1–16.9) nmol/L, respectively (Fig 2). When tested against DPP-4-related proteases, including DPP-2, DPP-8, DPP-9, PEP, and FAPα (Table 3), trelagliptin displayed IC_50_ values >100,000 nmol/L corresponding to >10,000-fold selectivity (Table 3). These data indicate that trelagliptin is a highly selective and potent DPP-4 inhibitor, about 4- and 12-fold more potent than alogliptin and sitagliptin, respectively.

**Table 2:** Inhibitory activity of trelagliptin in human, dog, and rat plasma.

Species of plasma	human	dog	rat
IC_50_ (nmol/L) (95% CI)	4.2 (4.1–4.3)	6.2 (6.0–6.4)	9.7 (8.0–11.8)

**Table 3:** Inhibitory activity of trelagliptin against DPP-4-related proteases.

Proteases	DPP-2	DPP-8	DPP-9	PEP	FAPα
IC_50_ (nmol/L)	>100,000	>100,000	>100,000	>100,000	>100,000

### Demonstration of competitive inhibition

**Fig 3:**
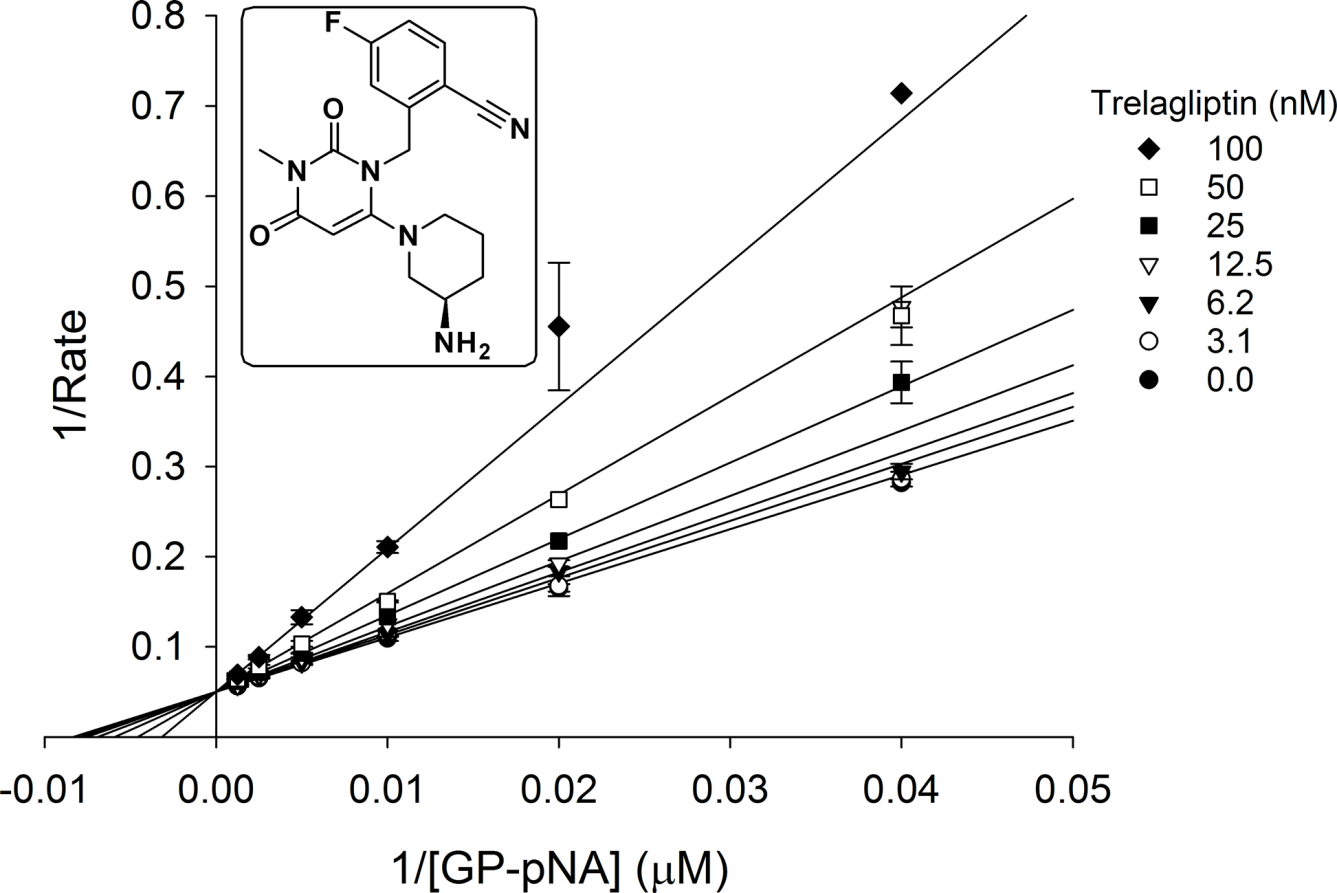
Double-reciprocal plot showing competitive inhibition of DPP-4 by trelagliptin. Initial rate (first 40 seconds only) was measured as described under Materials and Methods using a range of GP-pNA substrate concentrations bracketing the apparent Km value at several fixed trelagliptin concentrations.

Initial rate (first 40 seconds only) measured across a range of GP-pNA substrate concentrations bracketing the apparent 120 μmol/L Km value at several fixed trelagliptin concentrations and plotted in Lineweaver-Burk format (1/rate versus 1/[GP-pNA]) as shown in Fig 3, demonstrate that trelagliptin competes directly with substrate for binding at the DPP-4 active site, with an apparent K_i_ value of 61 nmol/L for the initial weak EI complex (see Fig 1).

### Slow-binding inhibition

**Fig 4:**
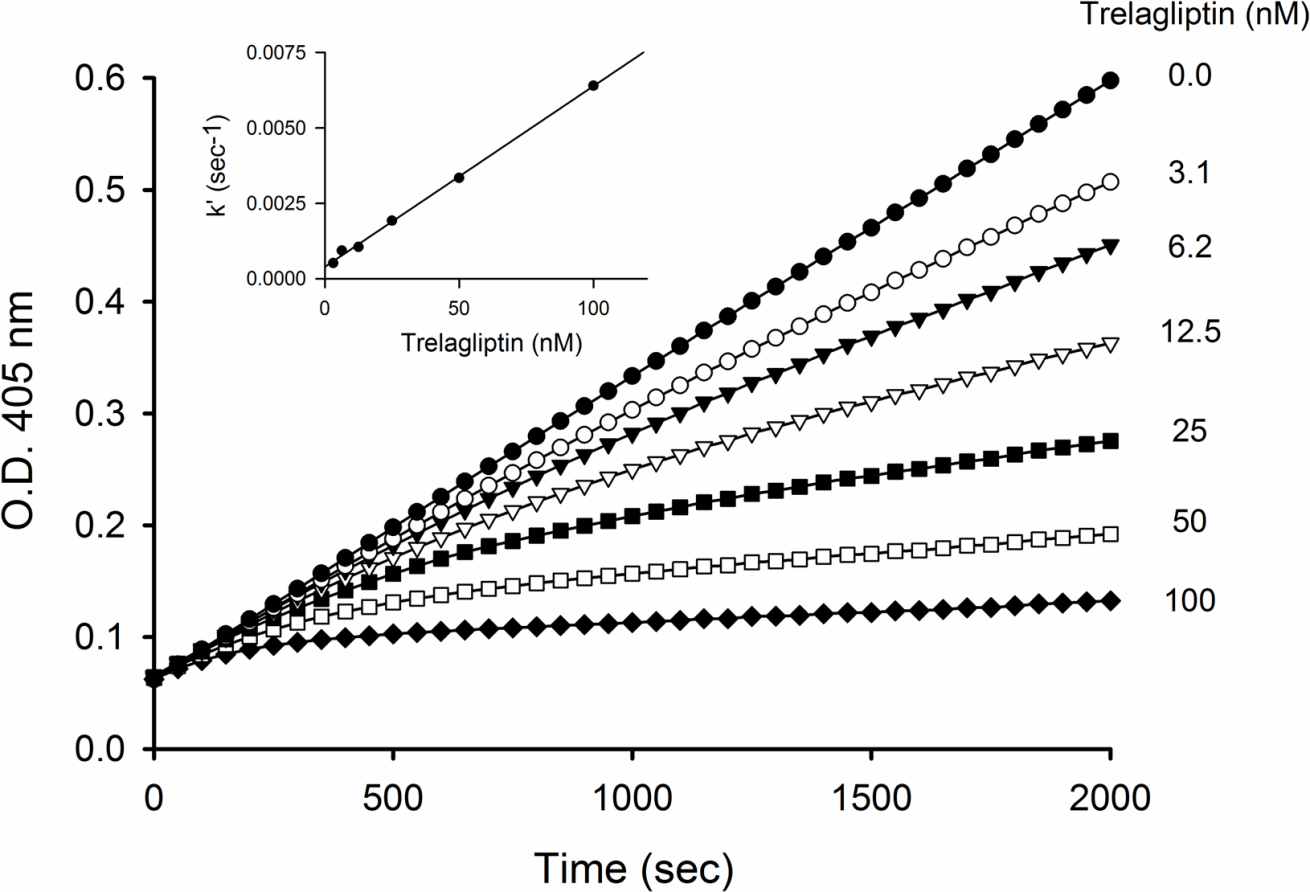
Time course of the reaction of DPP-4 in the absence or presence of different concentrations of trelagliptin. Progress curves at 405 nm for pNA generation were recorded over 2000 sec using a 10 sec interval. Reaction was initiated with 1 nmol/L DPP-4 in the presence of 400 μmol/L GP-pNA substrate (approximately 4x Km) and varying concentrations of trelagliptin. Inset: Replot of apparent association rate constant, k’, against trelagliptin concentration used to estimate k_on_’ from the slope according to Eq 7.

**Fig 5:**
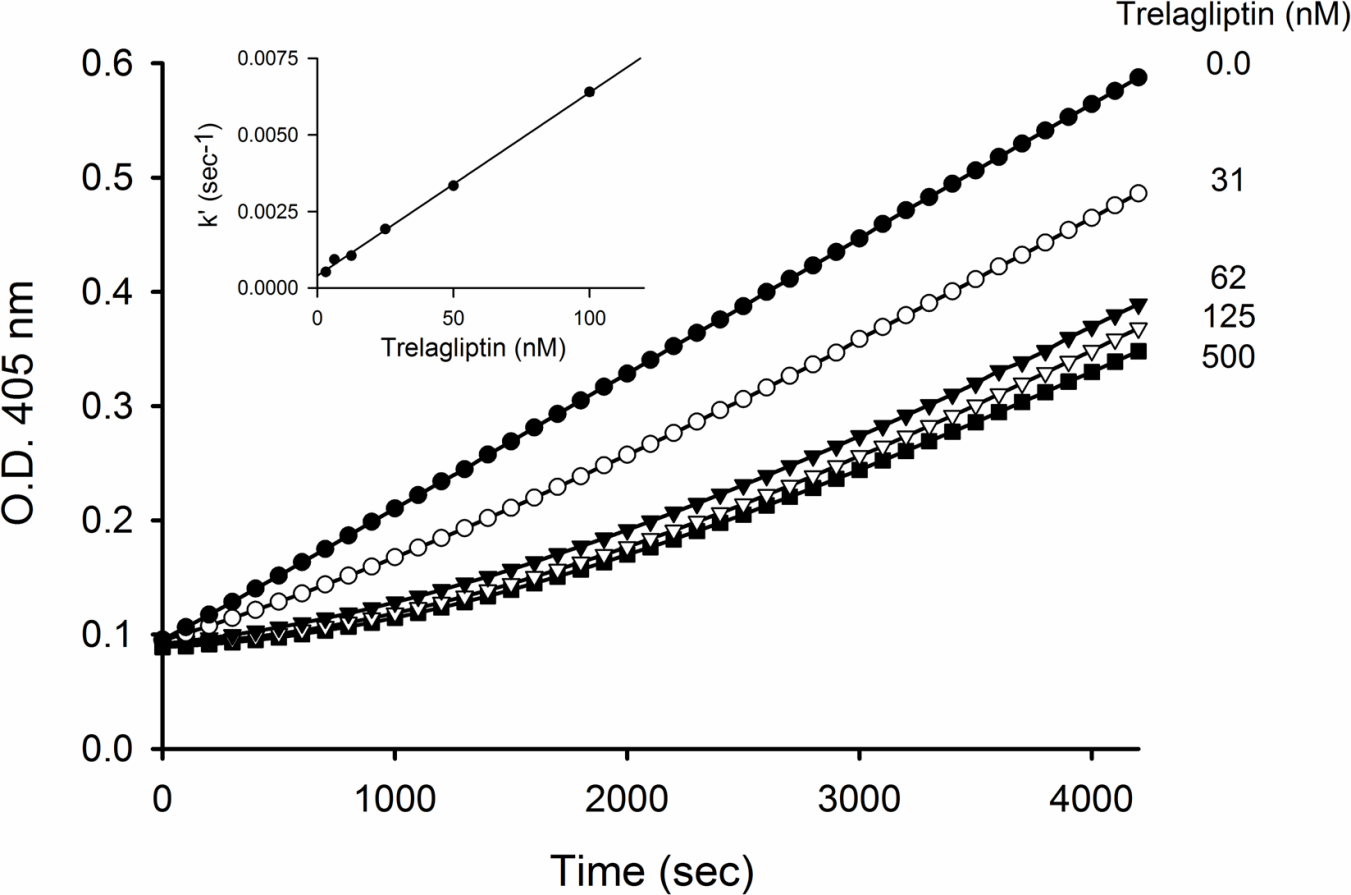
Time course of the recovery of DPP-4 activity following dissociation of trelagliptin from the preformed DPP-4-inhibitor complex. A preformed enzyme-inhibitor complex (where [DPP-4] = 50 nmol/L and trelagliptin concentration is as shown in the plot) was diluted 50-fold into a solution containing 2 mmol/L GP-pNA substrate (approximately 17x Km). Absorbance readings were taken every 10 seconds. Inset: Replot of the apparent dissociation rate constant, k’, against trelagliptin concentration used to estimate k_off_ from the Y-intercept according to Eq 7.

Deviation from linearity observed for reaction progress curves as the trelagliptin concentration was increased when either DPP-4 enzyme (onset of inhibition, Fig 4) or GP-pNA substrate (recovery of activity from preformed DPP-4-inhibitor complex, Fig 5) was added last to initiate the reaction, is consistent with slow-binding inhibition [10]. Kinetic analysis of both sets of progress curves provided estimates for k_on_ (Table 4) and k_off_ (Tables 4 and 5) that are internally consistent, and that correspond to a t_1/2_ ≈ 30 min for dissociation of trelagliptin from DPP-4. The 1.5 nmol/L value for K_i_* estimated as the k_off_/k_on_ ratio determined from the detailed kinetic analysis was furthermore consistent with the 1.0 nmol/L K_i_* value estimated from a Dixon plot of 1/*v*_*s*_ versus trelagliptin concentration at long reaction times (replot not shown), where the tight EI* complex makes the major contribution to the inhibition. The roughly 40-fold difference between K_i_ and K_i_* determined implies a K_isom_ (= k_2_/k_-2_) value of approxiately 40, reflecting a 40-fold tighter binding of trelagliptin in the tight EI* complex as opposed to the weak initial EI complex.

**Table 4:** Kinetic constants for trelagliptin determined from association progress curves.

parameter	Ki*(nmol/L)	k_on_ (M^-1^ sec^-1^)	k_off_ (sec^-1^)	EI* t_1/2_ (min)
trelagliptin	1.5 ± 0.1	(2.6 ± 0.1) x 10^5^	(4.0 ± 0.5) x 10^−4^	29

**Table 5:** Kinetic constants for trelagliptin determined from dissociation progress curves.

parameter	k_off_ (sec^-1^)	EI* t_1/2_ (min)
trelagliptin	(3.7 ± 0.5) x 10^−4^	31

Similar kinetic studies conducted using sitagliptin did not reveal any evidence for slow-binding inhibition (data not shown), indicating an upper limit for t_1/2_ < 2 min for dissociation of sitagliptin from DPP-4, which is consistent with the t_1/2_ ≈ 0.2 min value reported from surface plasmon resonance studies [16].

### DPP-4 crystal structure with trelagliptin

**Fig 6:**
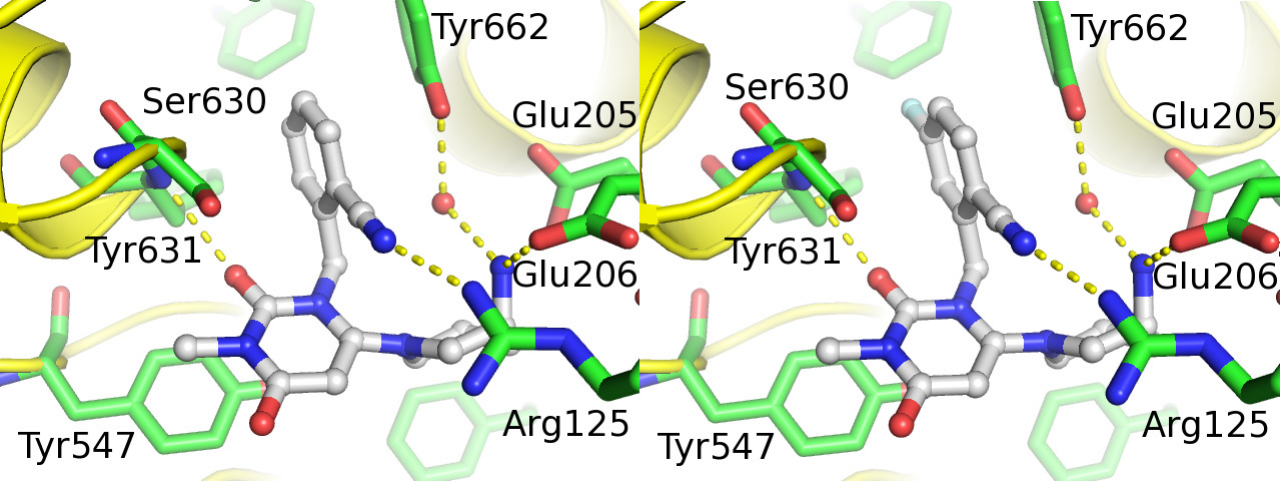
Comparison of x-ray crystal structures of inhibitors bound to DPP-4. Comparison of x-ray crystal structure of inhibitors bound to DPP-4 for alogliptin (left panel) and trelagliptin (right panel).

**Fig 7:**
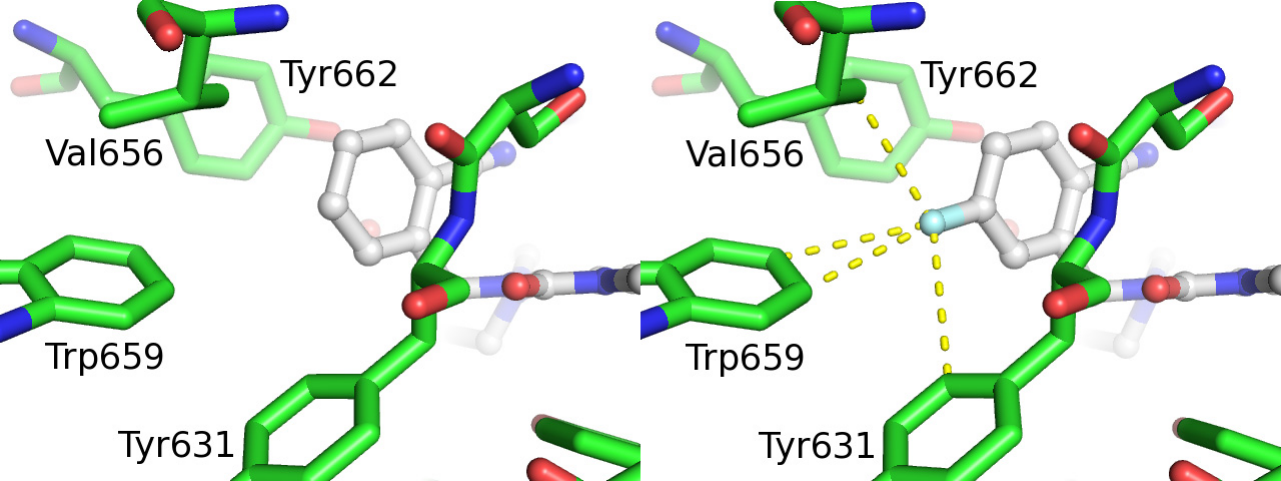
Potential fluorine atom interactions in trelagliptin x-ray crystal structure. Close-up showing potential differential interaction of F-atom of trelagliptin (right panel) with Trp659 residue in DPP-4 crystal structure as compared to H-atom of alogliptin (left panel).

Both trelagliptin and alogliptin were discovered with the aid of structure based drug design, and we were able to obtain co-complex X-ray crystal structures of both compounds bound to DPP-4. The co-complex structures of alogliptin and trelagliptin in the active site of DPP-4 are shown in Fig 6. Both structures show that the aminopiperidine forms a salt bridge to Glu205/Glu206, while the cyanobenzyl group effectively fills the S1 pocket (formed by Val656, Tyr631, Tyr662, Trp659, Tyr666, and Val711) and interacts with Arg125. The 2-position carbonyl participates in an important hydrogen bond with the backbone NH of Tyr631, and the uracil ring π-stacks with Tyr547. The single chemical structural difference between the two compounds is the presence of a fluorine atom at the 5-position of the cyanobenzyl group. Examination of the environment around the fluorine atom in the DPP-4 active site reveals the proximity of residues Trp659, Tyr631, and Val656 (Fig 7). Of these residues, only hydrogen atoms are within Van der Waals distance of the fluorine. These hydrogen atoms carry a permanent partial positive charge due to the position on the aromatic rings of Trp659 and Tyr631, and an inducible positive charge on the side chain of Val656. Fluorine carries a permanent partial negative change and so these atoms would be expected to produce an additional attractive force between ligand and protein, compared to a hydrogen atom at the 5-position. The magnitude of this attraction would be expected to be small but may be responsible for the 4-fold potency increase observed for trelagliptin versus alogliptin (Fig 2). The structures show that both compounds bind non-covalently to DPP-4.

## Discussion

Trelagliptin (SYR-472, zafatek) is a novel once-weekly DPP4 inhibitor that shows sustained efficacy by once-weekly dosing regimen in patients with type 2 diabetes. However, the underlying mechanism for this sustained efficacy remains to be clarified. In this study, we have characterized the *in vitro* properties for trelagliptin and compared those with alogliptin and sitagliptin.

Trelagliptin exhibits potent inhibition of human DPP-4 with IC_50_ values in the single digit nanomolar range, using either recombinant enzyme or a partially purified Caco-2 cell extract, that is approximately 4- to 12-fold more potent than either alogliptin or sitagliptin, respectively (Fig 2). The IC_50_ values for alogliptin and sitagliptin were consistent with those previously reported [17–20]. This enhanced potency carries over to human plasma when one compares the 4.2 nmol/L IC_50_ value for trelagliptin (Table 2) with the 10 nmol/L IC_50_ determined previously for alogliptin under similar experimental conditions (unpublished results). Thus, trelagliptin could exhibit a similar DPP-4 inhibition at lower plasma concentration compared with alogliptin.

X-ray diffraction data confirms that trelagliptin binds to DPP-4 in a pose (Fig 6) that is virtually identical to that seen for alogliptin, with the exception of the single fluorine atom at the 5-position of the cyanobenzyl group which may account for the slight potency advantage for trelagliptin. Unlike the covalent DPP-4 inhibitors, saxagliptin [21, 22] and vildagliptin [23, 24], where X-ray crystal structures show formation of a covalent imidate adduct between Ser630 and the cyano moiety of their cyano-pyrrolidine headgroup, X-ray results for both alogliptin and trelagliptin show no evidence for covalent complex formation. Initial velocity kinetic studies also confirm that DPP-4 inhibition by trelagliptin is reversible and substrate-competitive (Fig 3). In addition to enhancing binding affinity, the single fluorine substitution in trelagliptin also has the effect of slowing the dissociation rate for release of inhibitor from the enzyme by approximately 8-fold, if one compares the t_1/2_ ≈ 30 min for trelagliptin dissociation determined in this study (Tables 4 and 5) with the t_1/2_ ≈ 3.7 min value reported for alogliptin dissociation as determined by surface plasmon resonance [16]. Although the slower off-rate for trelagliptin compared to alogliptin contributes to more potent DPP-4 inhibitory activity, t_1/2_ for dissociation of trelagliptin from DPP-4 (≈ 30 min) is too short to entirely account for sustained efficacy of trelagliptin by once-weekly dosing.

What about other possible factors? No meaningful difference in plasma protein binding was observed between trelagliptin and alogliptin to explain differences in *in vitro* potency and *in vivo* efficacy (the binding of trelagliptin and alogliptin over the range of 0.1 to 10 μg/mL to human plasma protein were 22.1–27.6% and 28.2–32.3%, respectively (unpublished results)). In terms of microsomal stability using either rat or human microsomal preparations [18], trelagliptin (Compound 27j) showed slightly better stability than did alogliptin (Compound 27b), but again not sufficient to account for the much greater sustained efficacy. Likewise, comparing the pharmacokinetic and pharmacodynamic results obtained when trelagliptin was tested in dogs or cynomolgus monkeys [18] with those obtained using alogliptin [17], other than a slight potency advantage for trelagliptin, there was no indication from these preclinical studies that this compound would be amenable to a once-weekly as opposed to a once-daily dosing regimen. That conclusion was only revealed through human clinical trials [2, 5].

**Fig 8:**
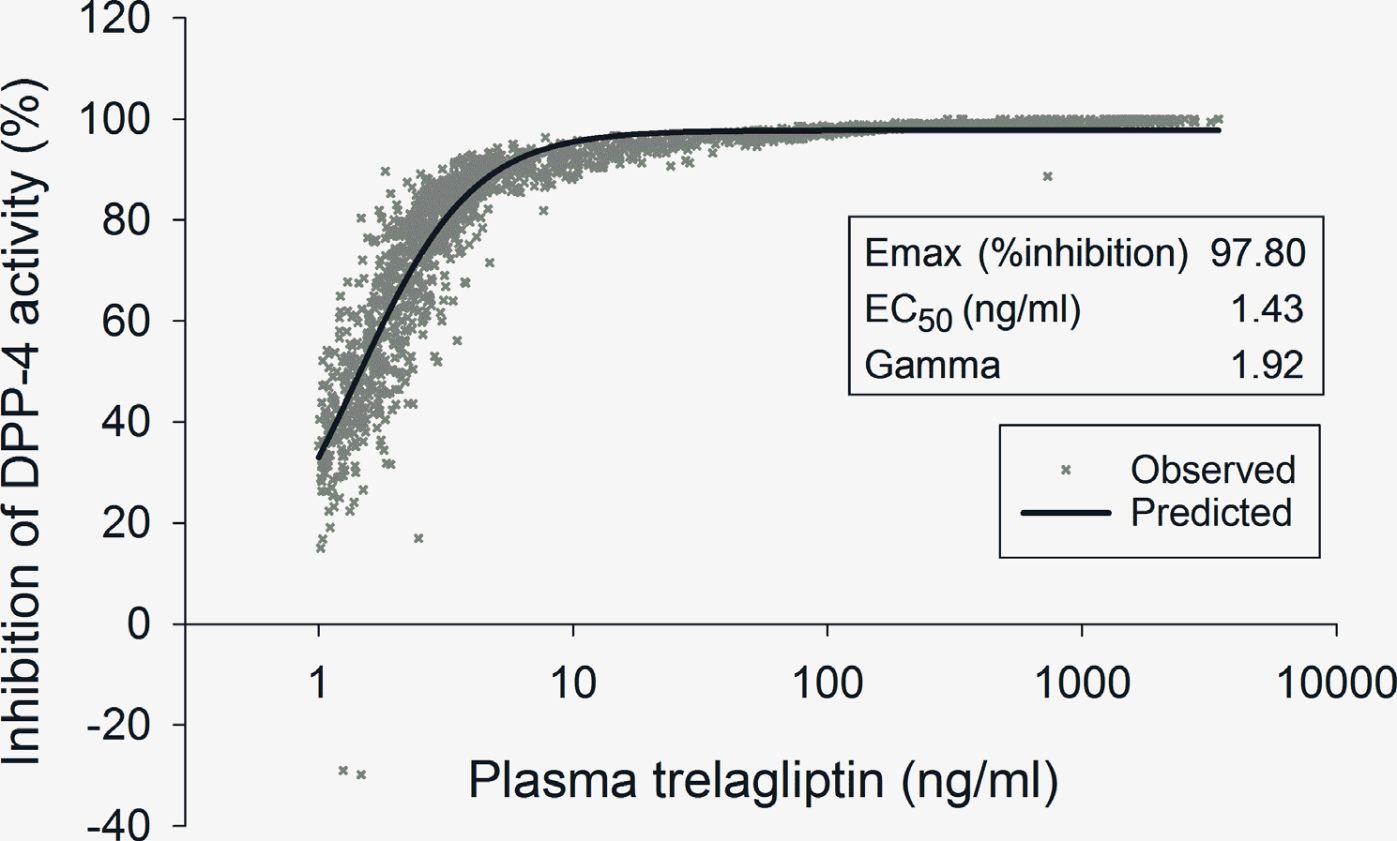
Relationship between trelagliptin pharmacokinetics and pharmacodynamics in T2DM patients in phase 2 dose-ranging study. Observed value plotted in “X” and predicted relationship between pharmacokinetics and pharmacodynamics by sigmoid Emax model is indicated by solid line. The plasma trelagliptin concentration estimated to yield 70% and 80% inhibition of human plasma DPP-4 activity was 2.31 ng/mL and 3.13 ng/mL, respectively.

In the clinical trial setting, trelagliptin showed sustained inhibition of DPP-4 activity until 7 days after dosing in the phase 2 dose-ranging study in patients with type 2 diabetes mellitus (T2DM) [2]. Further analysis of the results of this phase 2 dose-ranging study using a sigmoid Emax model indicated that the plasma trelagliptin concentration estimated to yield 50% and 70% inhibition of plasma DPP-4 activity was 1.43 ng/mL and 2.31 ng/mL, respectively (Fig 8). 1.43 ng/mL corresponds to about 4.02 nmol/L and is consistent with the IC_50_ value for DPP-4 inhibition in human plasma (Table 2). Moreover, in the previous phase 1 study [25], where single dose administration of trelagliptin (3.125–800 mg) was carried out in Japanese healthy volunteers, the plasma trelagliptin concentration [mean (SD)] at the 100 mg dose 168 hours after administration was 2.12 (0.679) ng/mL, and this value was almost the same as the plasma trelagliptin concentration estimated to yield 70% inhibition of plasma DPP-4 activity in phase 2 dose-ranging study as explained above (i.e., 2.31 ng/mL). These results indicate that the plasma concentration of trelagliptin even at 168 hours after 100 mg dosing is enough to sustain its pharmacodynamic effect, i.e., 70% inhibition of plasma DPP-4 activity, by weekly dosing of trelagliptin in T2DM patients. For futher insight into the high potency of trelagliptin, we refer to the similar pharmacokinetic/pharmacodynamic modeling performed for alogliptin [26], which is one of the once-daily DPP-4 inhibitors. Based on a simple Emax modeling, the estimated IC_50_ value of alogliptin was 6.6 ng/mL. The difference in estimated IC_50_ values (1.43 ng/mL [4.02 nmol/L] vs. 6.6 ng/mL [19.4 nmol/L]) between trelagliptin and alogliptin is consistent with the difference in IC_50_ values for human plasma DPP-4 inhibition *in vitro* (4.2 and 10 nmol/L for trelagliptin and alogliptin, respectively). These results suggest that the potent *in vitro* DPP-4 inhibitory activity of trelagliptin at least partially contributes to the *in vivo* efficacy of trelagliptin at lower plasma concentration at 7 days after administration. Of note, it has been shown that the efficacy of trelagliptin 100 mg weekly dosing is non-inferior to alogliptin 25 mg daily dosing in terms of reduction of HbA1c in T2DM patients [5].

In addition, no major safety concerns for trelagliptin have been confirmed and the overall safety profile of trelagliptin was similar to that of alogliptin in clinical studies [2, 5]. Selectivity for DPP-4 over other related serine proteases including DPP-8 and DPP-9 could be important to avoid potential adverse events. DPP-8 and DPP-9 inhibition was reported to be associated with multiorgan toxicities and immunotoxicity in rats and dogs and attenuation of human T-cell activation *in vitro* in some [27], but not other [28, 29] studies. In the present study, we demonstrated that trelagliptin shows > 10,000-fold selectivity for DPP-4 over other related serine proteases including DPP-8 and DPP-9. Moreover, no significant response (≥ 50% inhibition or stimulation) was observed at 10 μmol/L in an expansive selectivity counterscreen (47 enzymatic assays and 79 radioligand binding assays) carried out at MDS Pharma (data not shown). Thus, trelagliptin has a favorable target selectivity profile as a selective DPP-4 inhibitor.

## Conclusion

Trelagliptin is a novel once-weekly DPP-4 inhibitor that shows sustained efficacy by once-weekly treatment in T2DM patients. The *in vitro* data described in this paper demonstrated that trelagliptin is a potent and selective DPP-4 inhibitor. Kinetic analysis revealed that trelagliptin is a reversible, substrate-competitive and slow-binding DPP-4 inhibitor (t_1/2_ for dissociation ≈ 30 minutes). Furthermore, X-ray diffraction data indicated a non-covalent interaction between DPP-4 and trelagliptin. Potent DPP-4 inhibitory activity of trelagliptin may at least partially contribute to the sustained efficacy of trelagliptin.
